# Current Status of Vector-Borne Diseases in Croatia: Challenges and Future Prospects

**DOI:** 10.3390/life13091856

**Published:** 2023-09-01

**Authors:** Tatjana Vilibic-Cavlek, Natasa Janev-Holcer, Maja Bogdanic, Thomas Ferenc, Mateja Vujica Ferenc, Stjepan Krcmar, Vladimir Savic, Vladimir Stevanovic, Maja Ilic, Ljubo Barbic

**Affiliations:** 1 Department of Virology, Croatian Institute of Public Health, 10000 Zagreb, Croatia; 2 School of Medicine, University of Zagreb, 10000 Zagreb, Croatia; 3 Environmental Health Department, Croatian Institute of Public Health, 10000 Zagreb, Croatia; 4 Department of Social Medicine and Epidemiology, Faculty of Medicine, University of Rijeka, 51000 Rijeka, Croatia; 5 Department of Diagnostic and Interventional Radiology, Merkur University Hospital, 10000 Zagreb, Croatia; 6 Department of Obstetrics and Gynecology, University Hospital Center Zagreb, 10000 Zagreb, Croatia; 7 Department of Biology, Josip Juraj Strossmayer University of Osijek, 31000 Osijek, Croatia; 8 Poultry Center, Croatian Veterinary Institute, 10000 Zagreb, Croatia; 9 Department of Microbiology and Infectious Diseases with Clinic, Faculty of Veterinary Medicine, University of Zagreb, 10000 Zagreb, Croatia; 10 Department of Communicable Disease Epidemiology, Croatian Institute of Public Health, 10000 Zagreb, Croatia

**Keywords:** arboviruses, vector-borne bacteria, epidemiology, invasive mosquitoes, Croatia

## Abstract

Different vector-borne pathogens are present or have (re-)emerged in Croatia. Flaviviruses tick-borne encephalitis (TBEV), West Nile (WNV), and Usutu (USUV) are widely distributed in continental regions, while Toscana virus (TOSV) and sandfly fever viruses are detected at the Croatian littoral. Recently, sporadic clinical cases of Tahyna orthobunyavirus (TAHV) and Bhanja bandavirus infection and seropositive individuals have been reported in continental Croatia. Acute infections and serologic evidence of WNV, TBEV, USUV, and TAHV were also confirmed in sentinel animals and vectors. Autochthonous dengue was reported in 2010 at the Croatian littoral. Lyme borreliosis is the most widely distributed vector-borne bacterial infection. The incidence is very high in northwestern and eastern regions, which correlates with numerous records of *Ixodes ricinus* ticks. Acute human *Anaplasma phagocytophilum* infections are reported sporadically, but there are many records of serologic evidence of anaplasmosis in animals. Mediterranean spotted fever (*Rickettsia conorii*) and murine typhus (*Rickettsia typhi*) are the main rickettsial infections in Croatia. Human leishmaniasis is notified sporadically, while serologic evidence of leishmaniasis was found in 11.4% of the Croatian population. After the official eradication of malaria in 1964, only imported cases were reported in Croatia. Since vector-borne diseases show a growing trend, continuous monitoring of vectors is required to protect the population from these infections.

## 1. Introduction

Many vector-borne zoonotic pathogens are present or have (re-)emerged in Croatia in the past decades, including arboviruses, vector-borne bacteria, and parasites. Tick-borne encephalitis virus (TBEV) is endemic in Croatia; however, new natural micro-foci have been detected in some continental regions [1]. In 2010, two autochthonous dengue virus (DENV) infections were reported at the Croatian littoral [2], followed by the detection of the first human cases of West Nile virus (WNV) neuroinvasive disease in 2012 [3]. Usutu virus (USUV) is another neuroinvasive arbovirus that emerged in Croatia with the first human clinical cases recorded in 2013 [4]. Acute WNV and USUV infections as well as seropositivity were also continuously detected in sentinel animals [5,6]. Although the Toscana virus (TOSV) seroprevalence is high among residents of the Croatian littoral (33.6–53.9%) [7], the virus is still neglected since human clinical cases are detected only sporadically [8,9]. Sandfly fever Sicilian (SFSV) and Naples virus (SFNV) have also been confirmed serologically, mostly on the Croatian islands [10]. Rare Tahyna orthobunyavirus (TAHV) and Bhanja bandavirus (BHAV) infections have also been observed since 2018 [11]. Lyme-borreliosis (*Borrelia burgdorferi* s.l.) is the most widely distributed vector-borne bacterial zoonosis in Croatia. Human granulocytic anaplasmosis (*Anaplasma phagocytophilum*), Mediterranean spotted fever (*Rickettsia conorii*), and murine typhus (*Rickettsia typhi*) are reported sporadically [12,13]. In addition, sporadic cases of leishmaniasis have also been recorded, while anti-leishmania antibodies were detected in 11.4% (4.0–22.2%) of Croatian residents, with the highest seropositivity in central coastal Dalmatia [14]. Malaria was a major public health problem in Croatia until it was officially eradicated in 1964. No autochthonous cases have been reported since 1958; however, imported cases are recorded regularly [15].

In this regional review, the most important vector-borne zoonoses in Croatia in humans and animals (‘One Health’) are discussed. In addition, the distribution of the most common arthropod vectors, detection of zoonotic pathogens in vectors, and national vector monitoring programs are presented.

## 2. Literature Search

To collect data on vector-borne diseases and mosquito/tick records in Croatia, a literature search was conducted in the following electronic databases: PubMed, Web of Science, Medline, and Scopus with no limitations placed on the year of publication and language restriction. Books, dissertations, Master’s theses, and conference proceedings were also included. Additionally, the search was conducted in un-digitalized journal volumes belonging to library collections. The keywords searched were: arboviruses, TBEV, WNV, USUV, TOSV, TAHV, BHAV, sandfly fever, dengue, chikungunya, Zika, Lyme borreliosis, *B. burgdorferi* s.l., anaplasma, rickettsia, Mediterranean spotted fever, murine typhus, *Rickettsia conorii*, *Rickettsia typhi*, leishmania, malaria, invasive mosquitoes, *Aedes albopictus*, *Ae. japonicus*, ticks, *Ixodes ricinus*, *Haemaphysalis punctata*, distribution, epidemiology, One-Health, and Croatia. Once a comprehensive list of abstracts were retrieved and reviewed, any studies appearing to meet inclusion criteria were reviewed in full. All studies published by 20 June 2023 were included in the review.

## 3. Arthropod-Borne Viral Infections

### 3.1. Tick-Borne Encephalitis

**Figure 1:**
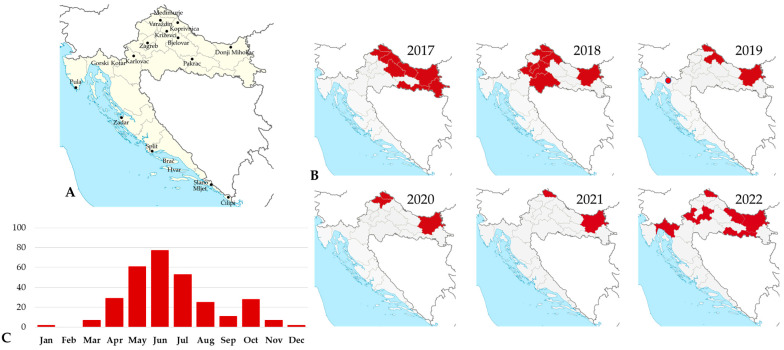
(**A**) Natural foci of tick-borne encephalitis in Croatia, 1960–2007 (places with documented human infections); (**B**) geographical distribution of human tick-borne encephalitis cases, 2017–2022 (shadowed areas represent counties with reported cases, red dot represents a cluster of TBE cases in a new micro-focus); (**C**) seasonal distribution of tick-borne encephalitis cases (number of reported cases by month).

TBEV is the most important tick-borne viral zoonosis in Europe. In Croatia, the disease was first described in 1953 in the northwestern continental region near Križevci (Stara Ves) [16]. In addition to the Pannonian natural focus, other foci in continental Croatia (Bjelovar, Pakrac, Koprivnica, Karlovac, Varaždin) as well as several smaller Mediterranean ones in the vicinity of Zadar, Pula, and the island of Brač have been detected since 1961 (Figure 1). The first clinical cases of tick-borne encephalitis (TBE) were described in 1968 on Medvednica mountain near Zagreb [17]. In 1991, a new natural TBE focus was detected in Gorski Kotar, a mountainous region between continental and coastal Croatia [18].

Seroprevalence studies on TBEV have been performed in humans and animals since 1960. From 1961 to 1964, Stara Ves was found to be an area with active virus circulation as suggested by high seroprevalence rates in both humans (32.14%) and animals (horses 86.96%, cattle 53.73%) [19]. In 1972, TBEV-neutralizing (NT) antibodies were detected in 47.50% of human and 59.49% of animal serum samples in the same area [20]. From 1962 to 1977, seropositivity was 22.43% in humans and 56.09% in sheep on the Island of Brač [21]. Furthermore, TBEV hemagglutination inhibiting (HI) antibodies were detected in 10–33% of inhabitants of northwestern, 2.9% of eastern, and 0.8% of northern Croatia. Lower seroprevalence rates (2.41–2.63%) were also detected in middle Dalmatia (Zadar and Split surroundings, Brač Island) and south Dalmatia, from Slano to Ćilipi [22]. In 1998–1999, TBEV antibodies were found in 1.15% of inhabitants of Hvar and 1.41% of inhabitants of Mljet islands [23]. In Međimurje (north-west Croatia), seroprevalence was 35% in 1997 compared to 16% in 2007 [24]. The natural foci of TBEV recorded from 1960 to 2007 are presented in Figure 1.

Human clinical TBE cases were continuously recorded in continental Croatian regions, with the highest number of infections in northwestern and eastern regions. In the period from 2017 to 2022, 73 cases were recorded in continental regions. A small outbreak following raw goat milk consumption was reported in 2019 in a new micro-focus in Gorski Kotar [1]. TBEV infection in Croatia showed a bimodal seasonality with a larger peak in the summer months (May–July) and a smaller one in autumn (September–October) (Figure 1).

In more recent studies (2017–2021), TBEV IgG antibodies were detected in horses from continental Croatian regions with seroprevalence rates ranging from 7.3% to 17.3%. The exposure of dogs in urban areas to TBEV and its possible correlation with human risk areas in Croatia were analyzed in 2016 since dogs as very popular pets live in close relationships with humans. A seropositivity of 3.65% was observed. The high prevalence of anti-TBE IgG antibodies in the dogs in Slavonski Brod and Bjelovar areas is in accordance with previous confirmation of these regions as moderate- to high-risk areas of TBE infections in humans [25]. Sheep were tested for TBEV antibodies in Vukovar-Srijem County (eastern Croatia) in 2022 showing a seropositivity of 9.0% [26].

TBEV was also detected in ticks (*Ixodes ricinus* and *I. hexagonus*) removed from red fox (*Vulpes vulpes*) carcasses hunted in endemic areas in northern Croatia in 2011. Furthermore, two TBEV-positive spleen samples from red deer were demonstrated [27].

Phylogenetic analysis of the Croatian TBEV strains detected in a human sample (2017), ticks, and deer spleen (2011) were shown to be closely related, all belonging to the European subtype TBEV [27,28].

### 3.2. West Nile Virus Infections

WNV is the most widely distributed mosquito-borne arbovirus in Europe. In Croatia, the first serologic evidence of WNV infection was reported in the 1970s. Seroprevalence rates of 4.9% (1970) and 0.28% (1974) were detected in the adult general population of Brač Island, while no schoolchildren from the same villages were seropositive in 1977 [29]. In addition, low seroprevalence rates were recorded in the Middle Adriatic (Nin—the estuary of Neretva River; 3.36%) and South Adriatic (Neretva estuary—Ulcinj; 0.77%) [22]. 

**Figure 2:**
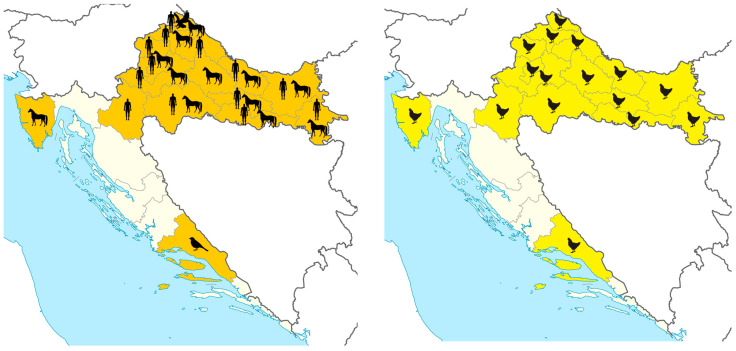
Distribution of acute WNV infections in humans and animals in Croatia, 2012–2022. Shadowed areas represent counties with reported infections; black silhouettes represent host species with WNV infections.

No seroprevalence studies were conducted thereafter until 2011. A pilot study (2011) showed WNV-neutralizing antibodies in 0.3% of the adult population [30]. The first outbreak of human WNV neuroinvasive disease (7 cases) was reported in 2012 in eastern counties bordering Serbia [3]. In a larger 2013 outbreak, a total of 20 cases were reported in three northwestern counties [4]. Sporadic cases were recorded from 2014 to 2016, followed by a small outbreak in 2017 (8 cases) and the largest outbreak so far in 2018 with 54 reported cases of neuroinvasive disease and 7 cases of WNV fever in 11 Croatian counties [6]. No human WNV infections were detected from 2019 to 2021. In 2022, a small outbreak of six patients with the neuroinvasive disease was reported in two northwestern and one eastern county [31] (Figure 2). After the first reports of human WNV infections, seroprevalence studies were conducted in counties with confirmed clinical cases showing seropositivity varying from 1.01% (2012–2013) to 3.2% (2020) [5].

Acute asymptomatic infections (IgM positive) in horses were continuously recorded from 2012 and preceded human cases. Most IgM-positive horses were in counties with human cases reported (Figure 2). In 2022, the first clinical WNV infection was observed in a horse presented with neurological signs [31]. Seroprevalence studies in horses detected WNV NT antibodies in 0.4% of randomly selected horses from eight Croatian counties tested between May 2001 and October 2002 [32]. Geographical distribution and number of seropositive horses (3.43%) detected during 2010–2011 indicated that WNV was quite prevalent in Croatia and that it was expanding from east to west [33]. Seroprevalence results from 2012 to 2021 showed high IgG seropositivity rates in continental regions; however, seropositive animals were also detected at the Croatian littoral [5].

During the largest WNV outbreak in 2018, brain tissues from 35 dead wild birds from families Passeridae (25), Accipitridae (4), Laridae (3), Anatidae (1), Ciconiidae (1), and Turdidae (1) were tested for the presence of WNV RNA. Fatal WNV infections were detected for the first time in Croatia in a female and a male goshawk (*Accipiter gentilis*) from the same aviary as well as serologic evidence of WNV infection in one buzzard (*Buteo buteo*) in northwest Croatia. One WNV-positive dead blackbird (*Turdus merula*) was also detected in Middle Dalmatia (Figure 2) [6]. 

The WNV activity in poultry was monitored using chickens (*Gallus gallus domesticus*) and turkeys (*Meleagris gallopavo domesticus*) as sentinels. The first serological evidence of WNV infection in poultry in Croatia was reported in 2013 in northwestern regions. From 2013 to 2020, serum samples from sentinel outdoor poultry were tested for the presence of WNV IgG antibodies. Serological surveys showed continuous WNV circulation with IgG seropositivity ranging from 1.8% to 22.9%. The majority of seropositive poultry was detected in continental Croatian counties [5] (Figure 2).

In a pilot study conducted on pet animals in 2021, WNV seropositive dogs (5.98%) and cats (4.35%) were detected in Zagreb and the surrounding area [34]. A seropositivity of 7.2% was observed in sheep (2022) from eastern Croatia [26].

Entomological surveys have been continuously performed since the first outbreak in 2012; however, all tested *Culex pipiens* and *Aedes albopictus* pools were negative for WNV RNA [35]. 

According to genetic lineages suggested by Rizzoli et al. [36], phylogenetic analysis showed that strains detected in humans (12 strains) and a goshawk belong to WNV lineage 2 [5,31]. The presented results confirmed active circulation and endemic presence of WNV in continental Croatia (Figure 2). So far, there are no reported human WNV cases at the Croatian littoral.

### 3.3. Usutu Virus Infections

**Figure 3:**
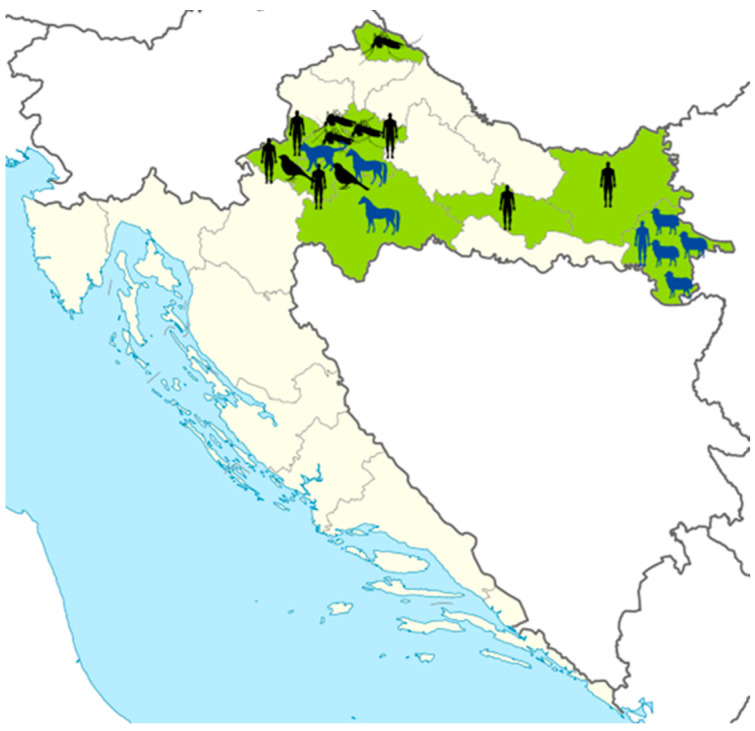
Distribution of Usutu virus in Croatia. Shadowed areas represent counties with reported infections; black silhouettes represent host species with acute clinical infections or USUV RNA detection; blue silhouettes represent species with serologic evidence of USUV infection (detection of neutralizing antibodies).

Although the number of USUV human infections is increasing, this virus is still neglected in many European countries. In Croatia, the first serologic evidence of USUV (detection of NT antibodies) was reported in 2011 in two seropositive horses from northwestern Croatia [37], followed by the detection of a seropositive human from eastern Croatia in 2012. The first three patients with the neuroinvasive disease were notified during the 2013 WNV outbreak in northwestern regions [4,38]. Three additional human cases were detected during the largest Croatian WNV outbreak in 2018 in the northwestern and eastern regions [6]. In the same year, USUV was confirmed in a dead blackbird (*Turdus merula*) in the Zagreb surrounding area. Additionally, one dead blackbird in Zagreb tested positive for USUV in 2022. In mosquitoes, USUV was detected in one pool each from 2016 (*Ae. albopictus*), 2017 (*Cx. pipiens* complex), 2018 (*Cx. pipiens* complex), and 2019 (*Cx. pipiens* complex) [35]. In 2022, the first USUV seropositive cat was detected in Zagreb [39]. In addition, in the same year, USUV NT antibodies were found in four sheep from Vukovar-Srijem County [26]. Strains detected in humans (2018), blackbirds (2018 and 2022), and mosquitoes (2018 and 2019) clustered within the USUV Europe 2 lineage [6,31,35]. The current distribution of USUV in Croatia (2011–2022) is presented in Figure 3. 

### 3.4. Toscana Virus Infections

TOSV is a sandfly-borne arbovirus distributed in many Mediterranean countries [40]. Seroprevalence studies indicated that TOSV is widely distributed among inhabitants of the Croatian littoral. In 2012, high seropositivity rates were detected in residents of Croatian islands (53.9%) and coastal areas (33.6%), but seropositive individuals (6.1%) were also recorded in the Croatian mainland [7]. However, human clinical cases of TOSV infections in Croatia are rarely reported. In 2007–2008, five patients with meningitis caused by TOSV were confirmed at the Croatian littoral [8]. Three cases of TOSV neuroinvasive disease (meningitis, meningoencephalitis) were also reported in 2018–2019 [9]. During the entomological study conducted in 2015, sandflies collected from five locations were tested for TOSV RNA. Co-circulation of TOSV lineages B and C was detected in *Phlebotomus neglectus* sandfly pools [41]. In Croatia, TOSV is not a routine consideration in the differential diagnosis of aseptic meningoencephalitis, therefore the true prevalence is not known, and the disease is probably underestimated and underreported.

### 3.5. Sandfly Fever

Sandfly fever is caused by different sandfly fever viruses transmitted by phlebotomine sandflies. Since sandfly fever reporting is not mandatory in Croatia, it is an underestimated and underreported disease. Large seroprevalence studies conducted in 1975–1976 showed the presence of SFSV and SFNV in both continental regions and the Croatian littoral. Very high seroprevalence rates were found on Brač and Hvar islands (62.1 and 59.4%, respectively). The SFNV was more frequent than SFSV and double SFNV/SFSV infections (Brač 46.44%, 4.74%, and 10.90%, respectively; Hvar 33.91%, 10.37%, and 15.12%, respectively) [42]. Serosurveys conducted in the 1980s showed an SFNV seroprevalence rate of 23.6% (5.9–45%) among residents of the Adriatic coast [43]. In a more recently conducted study (2017–2018), an overall seroprevalence rate of 2.3% and 3.3% for SFSV and SFNV, respectively, was found [10].

### 3.6. Tahyna Orthobunyavirus Infections

**Figure 4:**
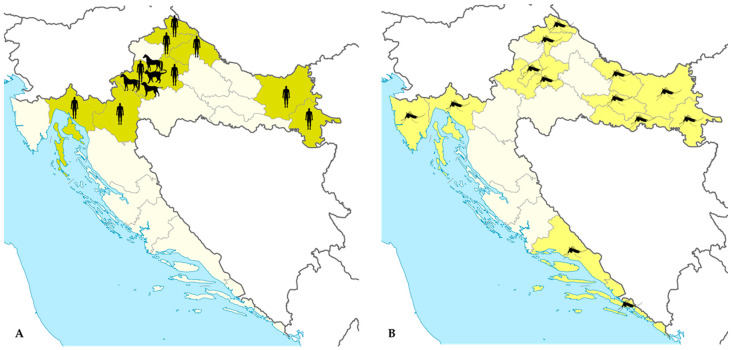
(**A**) Distribution of Tahyna orthobunyavirus in humans and animals in Croatia, 2017–2022. Shadowed areas represent counties with serologic evidence of TAHV infections (detection of neutralizing antibodies); black silhouettes represent host species seropositive to TAHV; (**B**) Counties with large presence of *Aedes vexans* mosquitoes.

TAHV is a mosquito-borne widely distributed arbovirus. However, due to a lack of commercially available diagnostic tests, this disease is still neglected and underreported. The first serologic evidence of TAHV in Croatia dates to the 1970s. A few seroepidemiological studies showed TAHV antibodies in 7.9% of inhabitants of northeast Croatia and 0.2–1.47% of inhabitants of the Croatian littoral [22,23]. In addition, TAHV HI antibodies were detected in the serum samples of free-ranging European brown bears (*Ursus arctos*) collected within the Plitvice Lakes and Risnjak National Parks (1984–1988) [44]. There were no data on the TAHV circulation thereafter until 2017. A recently conducted Croatian study (2017–2021) detected TAHV NT antibodies in 10.1% of patients with unsolved neuroinvasive disease who developed symptoms during the arbovirus transmission seasons. Recent TAHV infection was suspected in two patients presenting with meningitis, as suggested by the detection of TAHV NT antibodies in the cerebrospinal fluid (CSF). Seropositive patients were mainly residents of floodplains along the rivers in continental Croatia; however, sporadic infections were also confirmed in the coastal region [11]. From 2020 to 2022, a study of the seroprevalence of TAHV was conducted in humans, animals, and mosquitoes in urban areas of Zagreb and its surroundings. The prevalence of TAHV NT antibodies was 3.7% in asymptomatic individuals, 29.6% in horses, and 11.7% in pet animals (dogs, cats). None of the tested *Aedes vexans* mosquito pools tested positive for TAHV RNA [45]. The geographic distribution of TAHV seropositive humans and animals in Croatia is presented in Figure 4.

### 3.7. Bhanja Bandavirus Infections

BHAV is a neglected tick-borne bunyavirus. In Croatia, BHAV was isolated in 1974 from *Haemaphysalis punctata* ticks collected from sheep on Brač Island [46]. The first human laboratory infection was reported in 1974, followed by two additional laboratory infections in 1977 [47]. In 1975, BHAV was retrospectively diagnosed in a patient with meningoencephalitis and spastic quadriparesis in Zagreb [42]. Seroepidemiological surveys conducted after the virus isolation (1975 and 1977) showed BHAV HI antibodies in 31.54% of the inhabitants of Brač Island. Furthermore, while studying the potential virus reservoirs, serologic evidence of BHAV infection was confirmed in sheep from the same area [29]. The other study conducted in 1975 found BHAV-NT antibodies in 35.8% (11.6% to 61.3%) of the population from eight villages of Brač Island [48]. BHAV HI antibodies were also found in 2.2% of residents of the Middle Dalmatia (islands around Zadar) and 1% of the residents of Hvar Island as well as in 7.1% of the inhabitants of northern Croatia [22]. The prevalence of BHAV was not investigated thereafter. From 2020 to 2022, a study was conducted among 254 patients with neuroinvasive disease with unknown etiology. BHAV NT antibodies were detected in the CSF samples of two patients indicating a recent BHAV infection. Furthermore, BHAV NT antibodies were found in serum samples of additional 51 patients (the overall seroprevalence 20.8%), suggesting the presence of this virus in Croatia [49]. 

**Figure 5:**
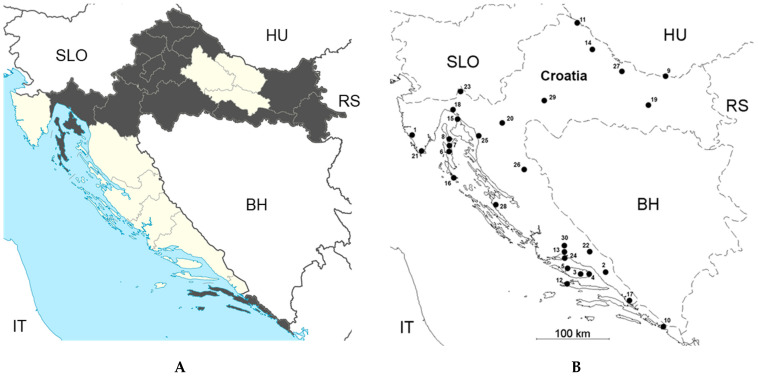
(**A**) Counties with reported Bhanja virus seropositive patients, 2020–2022 (shadowed areas; (**B**) places with *Haemaphysalis punctata* tick records (numbers represent locality labels).

The *H. punctata* tick is the main vector of BHAV in Croatia and was recorded on ten different host species. It is a three-host tick with a natural life cycle of one to three years. During its immature stages, its hosts are small mammals, birds, and lizards. Adults mainly feed on wild and domestic ungulates and rarely on humans. Larvae are active in the summer months from June to September, while nymphs have a bimodal activity during the season with peaks from April to October and December to February. The adults also have a bimodal activity with peaks from March to June and September to November [50]. Records of *H. punctata* in Croatia mainly correspond to the period of seasonal dynamic mentioned in the literature data for this species. However, depending on the geographic region, the seasonal dynamic of *H. punctata* varies. *Haemaphysalis punctata* is adaptable to different climatic conditions and because of that inhabits various habitats, ranging from cold to mild and humid to dry [49]. In Croatia, *H. punctata* was recorded in localities in all three biogeographic regions (Mediterranean, Alpine, and Continental), and has been recorded in 36 localities covering the 30 fields on the UTM grid (Universal Transverse Mercator coordinate system) of Croatia [27,51,52,53,54,55,56,57,58,59] (Figure 5, Table 1).

The geographic distribution of BHAV seropositive patients and the distribution of *H. punctata* ticks is presented in Figure 5.

### 3.8. Dengue, Chikungunya, and Zika Virus Infections

DENV, chikungunya (CHIKV), and Zika viruses (ZIKV) represent significant public health problems for travelers. A seroepidemiological study conducted in 1980 in a limited area of northeastern Croatia proved the presence of antibodies to DENV types 1 and 2 in 2.1% and 3.9% of healthy young inhabitants [60]; however, no cases of dengue were registered until 2010. After health professionals were notified of a DENV infection in a German tourist acquired in Croatia, the second case of autochthonous dengue was diagnosed in a resident of the Pelješac peninsula (South Dalmatia), the same region where the first patient had stayed. In the same area, 15 individuals showed serologic evidence of recent DENV infection [2]. A seroprevalence rate of 0.59% was found during the seroepidemiological study conducted in 2011–2012. Seropositivity varied with the highest seroprevalence rate (2.21%) in Dubrovnik-Neretva County where autochthonous dengue cases were recorded in 2010. There have been regular sporadic imported dengue infections in travelers returning from endemic areas [3]. A seroprevalence study on CHIKV conducted among inhabitants of the Croatian littoral (2011–2012) showed a seroprevalence rate of 0.9% (0.5–1.8%) [61]. Autochthonous chikungunya and Zika virus infections were not recorded, but sporadic imported cases have been continuously reported since 2016 [62,63].

## 4. Vector-Borne Bacterial Infections

### 4.1. Lyme Disease (Borrelia burgdorferi *s.l.*)

**Figure 6:**
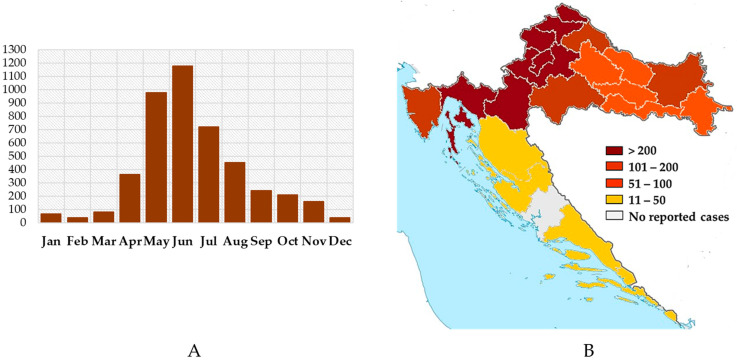
Seasonal and geographic distribution of Lyme borreliosis in Croatia (2011–2020): number of reported cases by month (**A**) and county (**B**).

Lyme borreliosis is the most common tick-borne zoonotic disease in Europe [64]. In Croatia, *Borrelia burgdorferi*, *B. afzelii*, and *B. garinii* were isolated in 1992, 1998, and 2002, respectively [65]. Lyme disease is reported in all Croatian counties; however, the disease is more frequent in continental regions. Seroprevalence studies in the 1990s showed IgG seropositivity rates of 9.7% in the general population and 42.9% in forestry workers. A significant difference in the seroprevalence between individuals from the endemic area (Koprivnica; 44%) and the non-endemic area was observed (Zagreb; 8%) [66]. In addition, low seropositivity was documented in the Gorski Kotar and the neighboring littoral area. The seroprevalence was 4.7% in the forestry workers, 3.0% in the general population of Gorski Kotar, and 2.7% in the general population of the littoral area. Furthermore, 40% of hunting dogs from the Gorski Kotar were IgG seropositive [67]. In high-risk areas, seroprevalence of up to 44% was recorded in the general population [66]. According to data from the Reference Center for Epidemiology Croatian Ministry of Health (Croatian Institute of Public Health), 400 to 800 cases of borreliosis are recorded annually. The incidence is highest in northwestern and eastern regions (Figure 6). Cases showed seasonal distribution which overlap with the tick activity (a large peak in spring and a smaller one in autumn).

**Figure 7:**
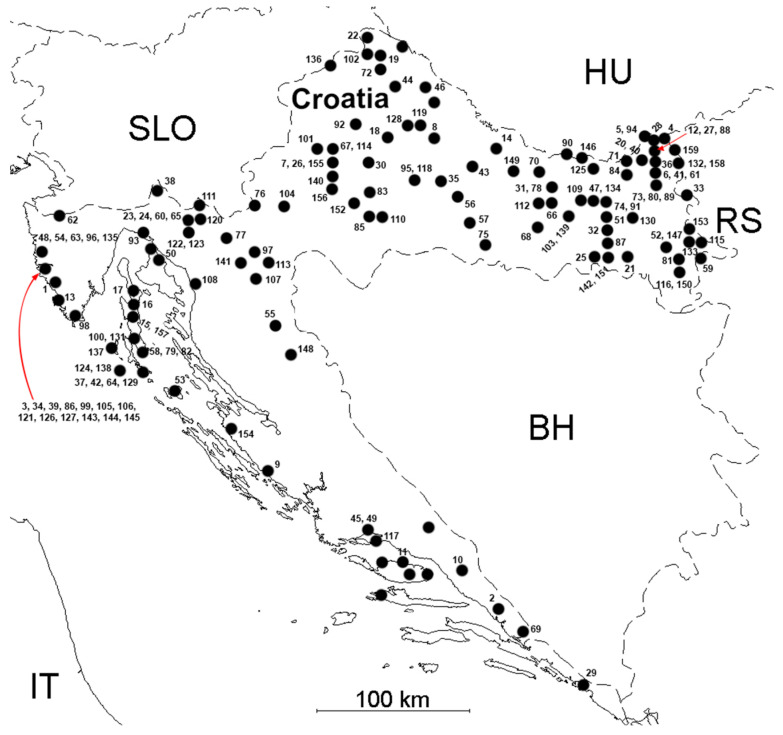
Distribution of *Ixodes ricinus* ticks in Croatia (black dots represent places with records). Geographic coordinates (altitude-latitude, UTM) are presented in the Supplementary Table S1.

*Ixodes ricinus* ticks play an important role in the spreading of borreliosis in the Croatian inland. The distribution of *I. ricinus* ticks in Croatia is presented in Figure 7. Records of ticks correlate with the number of reported human cases. *Ixodes ricinus* was recorded in localities in all three biogeographic regions (Mediterranean, Alpine, and Continental), and has been recorded in 207 localities covering the 159 fields on the UTM grid (Universal Transverse Mercator coordinate system) of Croatia [27,51,54,55,57,58,68,69,70,71,72]. Many different wild and domestic animals serve as hosts for *I*. *ricinus*. Twenty-seven hosts were recorded in Croatia. The primary habitats of *I. ricinus* are humid scrublands and deciduous or mixed forests [73]. Activity periods during the year are bimodal with activity peaks in spring and in autumn. *I. ricinus* is considered the most important vector of many zoonotic viruses and bacteria in Europe [74].

In 1996, a high prevalence (45%) of *B. burgdorferi* s.l. within the tick population collected in endemic regions of northern Croatia has been recorded [75]. In a recent study (2019–2021), ticks were collected at three localities in continental Croatia (Medvednica and Papuk) and an alpine biogeographic region (Gorski Kotar). *Ixodes ricinus* species predominated (99.83%). *Borrelia burgdorferi* s.l. was detected in 3.7% of ticks collected in the continental region and 20% of ticks collected in Gorski Kotar. Sequencing of the ospA gene demonstrated the presence of the *B. burgdorferi* s.s. genotype [72].

*Borrelia burgdorferi* s.l. antibodies were detected in 0.3% of dogs tested from 2012 to 2016 (0.7% from continental and 2.2% from coastal region) [76] and 39.4% of horses tested in 2016. The highest seroprevalence (40.0–47.1%) was observed in northwestern counties with a high incidence of human infections [77]. Antibodies to *B. burgdorferi* s.l. were also documented in roe deer and hare from the endemic region of northwest Croatia [78].

*Borrelia afzelii* was isolated in 2006 from the CSF of a patient with neuroborreliosis [65]. Additionally, *B. afzelii* and *B. garinii* were detected in 64.3% and 3.6% of skin biopsies of erythema migrans patients tested in the period from 2008 to 2010 [79]. *Borrelia miyamotoi* was detected in 3.7% of small rodents (*Apodemus agrarius*, *A. flavicolis*, and *Sorex araneus,* trapped between 2003 and 2011 in northwestern (Ivanić Grad) and eastern Croatia [80]. So far, there are no recorded human infections caused by *B. miyamotoi* in Croatia. 

### 4.2. Anaplasmosis (Anaplasma phagocytophilum)

The *Anaplasma phagocytophilum*, *A. platys*, *A. marginale*, and *A. bovis* are re-emergent pathogens that cause tick-borne diseases in humans and animals [81]. Human infections are confined to *A. phagocytophilum* only. The first cases of human granulocytic anaplasmosis (HGA) in Croatia were identified in 1998 in continental Koprivnica-Križevci County [12]. Since 2000, several seroepidemiological studies have been conducted to identify HGA, especially in the endemic areas of tick-transmitted diseases. One of the first published studies was conducted in 2003. Tick-borne zoonoses were analyzed in eastern Croatia (Vukovar-Srijem, Osijek-Baranja, and Brod-Posavina Counties) among 102 individuals who reported a history of tick bites. Seven serum samples were positive for HGA—four patients were asymptomatic and three were hospitalized with symptoms of TBE [82]. The potential anaplasmatic etiology of febrile illness after a tick bite during the 7-year period (1998–2004) was investigated in Koprivnica-Križevci County [83]. Out of 132 included patients, 8 (6%) of them were diagnosed with anaplasmosis; 3 patients had a confirmed and 5 patients had a probable infection. In a study from 2015, out of 425 tested patients, 160 (37.65%) had anti-*A. phagocytophilum* antibodies. Acute HGA was detected in three patients (0.01%), and high levels of IgG (titers ≥ 256) probable for HGA were found in 40 patients (0.09%) [12]. In the period 2017–2018, patients with neuroinvasive disease and a history of a tick bite from continental regions were tested for *A. phagocytophilum*. Acute anaplasmosis was not detected; however, IgG antibodies were detected in 26.7% of patients indicating previous exposure to *A. phagocytophilum* [84]. 

*A. phagocytophilum* has also been found in dogs, cats, horses, cattle, small ruminants, and forest animals [12,76,81,85,86,87]. Seroprevalence rates among dogs varied from 0.28 to 6.21% (Table 2) [85,88]. In 2017, *A. phagocytopilum* was detected by PCR in 3 out of 1080 apparently healthy Croatian dogs; two cases (0.8%) were detected in the continental region, whereas one case (0.14%) was detected in the Dalmatia region at the Croatian littoral [81]. A large-scale serological study of vector-borne pathogens (2012–2016) divided tested dogs into three categories: asymptomatic, suspected, and deceased dogs. Overall, 4.5% of dogs were seropositive to *A. phagocytophilum*: 5.4% of the asymptomatic, 3.4% of the suspected, and 3.2% of the deceased group [76]. In 2019, *A. phagocytophilum* antibodies were detected in 3.73% of pets with outdoor habitation and freedom of movement on the Istrian peninsula [89]. In 2017, the first case of equine anaplasma infection was detected using PCR in northeast Croatia (Slavonia region) in a 14-year-old Croatian warmblood mare [87]. 

In Europe, *A. platys* was primarily detected in dogs [81,90]. Only one recent Croatian study investigated the prevalence of *A. platys* among randomly selected healthy dogs. The overall prevalence rate using PCR was 2.5%. *A. platys* was most prevalent in the North Adriatic region (7.1%), followed by Dalmatia (2.0%), and the continental region (1.6%). *A. platys* was mainly distributed along the Croatian coast, the big cities in Dalmatia (Split), and the North Adriatic region (Pula) [81]. 

In the European region, *A. marginale* is confined to the Mediterranean and central European countries [86,91]. *Dermacentor reticulatus* tick has been identified as a vector in Europe; however, *A. marginale* may also be transmitted by biting flies, farm equipment, and needles contaminated with blood. Since the origin of the pathogen is undefined, there is a presumption among domestic veterinarians that the pathogen has been present in the former Yugoslavia since the 1930s. The seroprevalence rate of *A. marginale* in Croatia has yet to be determined, and sporadic cases of infected cattle have been reported on micro-locations [86]. 

*A. bovis* and *A. centrale* have been previously reported in cattle from Italy, and roe deer and red deer in Spain [86]. Ticks of *Rhipicephalus* spp. and *Haemaphysalis* spp. may transmit *A. bovis*, while *A. centrale* is presumably transmitted by *Rhipicephalus simus* [86,92]. The presence of *A. bovis* was detected in Croatian mouflons, roe deer, red deer, and red foxes [86]. To our knowledge, *A. centrale* has yet to be identified in Croatia.

### 4.3. Rickettsioses

During World War II, epidemic typhus outbreaks (*Rickettsia prowazekii*) were common in the territory of former Yugoslavia. Between 1945 and 1969, a total of 2794 human cases of epidemic typhus were reported in Croatia. Since 1969, Croatia appears to be free of epidemic typhus, and the last case of Brill–Zinsser disease was recorded in 2008 [93]. Other rickettsioses have been recorded continuously throughout the Croatian littoral. Mediterranean spotted fever (MSF; *Rickettsia conorii*) is the most frequent human rickettsial infection in Croatia, followed by murine typhus (*Rickettsia typhi*). Human MSF and murine typhus cases have been predominantly observed along the eastern Adriatic coast from Zadar to Dubrovnik. *Rickettsia akari*, a causative agent of rickettsialpox, was isolated from the blood of a patient in Zadar in 1991, but no further cases of rickettsialpox were reported. The subsequent seroepidemiological survey showed that 24.8% of individuals living in the Zadar area and adjacent islands had antibodies to *R. akari*. However, due to possible cross-reactivity between spotted fever group rickettsiae, such a high seroprevalence should be interpreted cautiously [94].

Between 1982 and 2002, a study on MSF was conducted among patients hospitalized at the infectious disease department in Split-Dalmatia County and included two regions: littoral (including towns and their suburbs) and islands (Vis, Hvar, Brač, and Šolta). The majority of patients (71.4%) originated from the islands and coastal areas. MSF showed seasonal distribution with the most cases (81.7%) occurring from July to September and a peak incidence in August (42.1%). Murine typhus was detected in 57 patients. Like MSF, 66.7% of patients originated from islands and coastal areas. The disease was observed all year round [13]. Serological testing among patients with febrile disease and rash (2009–2012) showed IgG antibodies to *R. conorii* in 29% of patients, *R. typhi* in 8% of patients, and 4% were seropositive to both rickettsia. Seropositive individuals were detected in the coastal areas, eastern and northwestern Croatia [95]. 

Several rickettsial species (*R. slovaca*, *R. aeshlimannii, R. helvetica*, and *R. raoultii*) were detected in ticks collected in different ecological regions of Croatia. In 2000, rickettsial DNA was detected in 12.7% of ticks removed from sheep, goats, and cattle in the wider area of Split. *R. helvetica* (10%) and *R. slovaca* (2%) were detected in *Dermacentor reticulatus* ticks (2007) collected on meadows in two different locations near Čakovec, between the Drava and Mura [96].

## 5. Vector-Borne Parasitic Infections

### 5.1. Leishmaniais

Up to 2020, leishmaniasis has been documented in 20 European countries while the Mediterranean Basin is an endemic and most affected region [97,98]. The most important pathogens in Europe are *L. major*, *L. tropica*, *L. donovani*, and *L. infantum* [98]. 

**Figure 8:**
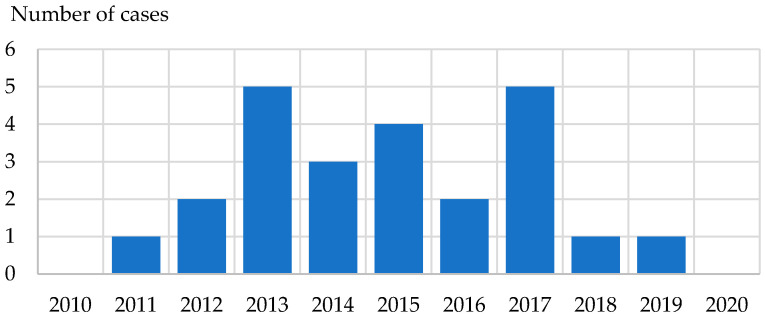
Reported cases of human leishmaniasis in Croatia, 2010–2020.

Since 1911, visceral leishmaniasis (VL) has been known among the coastal Croatian population; however, the first officially reported cases date from 1930 [99,100,101]. During that year, 9 cases were recorded in the city of Split, 8 in the city of Makarska, and 27 in villages surrounding the city of Dubrovnik [99,101]. In the Dalmatia region, between 1931 and 1948, there were 248 confirmed cases of VL from the cities of Split, Makarska, Dubrovnik, the island of Korčula, and Supetar districts [99]. Until the 1950s, 90.4% of patients were children under 10 years [99,102]. From 1954 to 1974, 70 individuals were diagnosed with VL, and from 1975 to 1991 only 3 [99,103]. In the war, and post-war period (1992–1997), 11 cases of the disease were registered [103]. Moreover, VL was detected in 23 people between 1994 and 2006 [104]. In general, in the past two–three decades, VL was considered to be a pediatric disease since 64.3% of cases include children under 10 years of age [102,103]. The first CL case in Croatia was reported in 1945, with a further 201 cases from 1945 to 1957, mainly in the cities of Split and Makarska [99,101]. Since the 1960s, only sporadic cases of CL have been detected in central and southern Dalmatia [101]. Between 1974 and 1993, only 18 individuals were diagnosed with CL, whereas from 1994 to 2006, 12 cases were reported with an estimated 3 cases per year [99,104]. Sporadic leishmania cases (24 patients) were reported from 2010 to 2020 (Figure 8). Seroprevalence studies on human leishmaniasis in Croatia are scarce. Only one study (2013) evaluated the prevalence of *L. infantum* IgG antibodies among healthy asymptomatic inhabitants of the Croatian littoral and mainland. Using ELISA, anti-*L. infantum* IgG antibodies were found in 11.4% of participants with seroprevalence rates varying between 0.0% in Brod-Posavina County (mainland) to 22.2% in central Dalmatia (littoral) [14]. 

The first investigations of phlebotomine sandflies in former Yugoslavia started in 1900; however, more detailed research was not initiated until 1928 [105]. According to a recent study (2020), a great number of sandfly species have been documented in former Yugoslavia since 1900: *Phlebotomus papatasi*, *P. sergenti*, *P. neglectus (major)*, *P. perfiliewi*, *P. simici*, *P. perniciosus*, *P. tobbi*, *P. balcanicus*, *P. mascittii*, *P. alexandri*, *P. kandellaki* (unpublished data), *Sergentomyia minuta*, and *S. dentata* [97]. In a study conducted on the Istrian peninsula near illegal waste sites (2015), all of the female sandflies tested negative for leishmania. Leishmania DNA was confirmed in the splenic tissue of one rat (*Rattus rattus*) near the city of Umag (Croatia) [106]. In addition to rats, known reservoirs of leishmaniasis are dogs and jackals (*Canis aureus*) [104].

In Croatia, the first cases of canine leishmaniasis were from the city of Split and date from 1931 [107]. Further studies in the coastal region on stray dogs (1931–1934) discovered 7.6% positivity, and studies on stray and domestic dogs (1950) a 5.8% positivity of leishmaniasis [99]. ELISA screening of 51 hunting dogs from the Konavle district between 1997 and 2000 resulted in 6% of positive samples [107]. In 2003, a large-scale screening study on the leishmania seroprevalence among dogs from the city of Split and 12 surrounding villages was conducted. The seropositive dogs were identified in the city of Split and 8/12 surrounding villages with mean seropositivity rates of 14.7% in Split and 7.1–42.8% in surrounding villages [108]. Further studies in dogs (2005) detected 7.9% of positive serum samples, while using PCR, 80.5% of samples were positive for *Leishmania* spp. in 2006. In 2008, a seroprevalence study was conducted among symptomatic and asymptomatic dogs from endemic regions (Split-Dalmatia, Šibenik-Knin, and Dubrovnik-Neretva Counties). Results showed that 40.4% and 14.2% of dogs were seropositive, respectively [107]. The other study from 2008 evaluated 74 apparently healthy dogs from Split-Dalmatia County and Šibenik-Knin County on the presence of anti-*Leishmania* spp. antibodies using ELISA. Authors revealed 9/29 positive dog samples in Split-Dalmatia County and 1/45 positive samples in Šibenik-Knin County, with a total of 13.5% of seropositive dogs [109]. In the most recently published study (2017), 1.38% of examined dog serum samples were positive for *L. infantum*, all from the city of Dubrovnik [88]. In addition to dogs, in 2003, *L. infantum* was confirmed in wild canids (*Canis lupus*). In the following study, samples of lymphatic tissue from 71 deceased wolves were collected during a 14-year period and 11 (15%) wolves from the Dalmatia, Lika, and Gorski Kotar regions tested positive for *L. infantum* using PCR [107]. 

### 5.2. Malaria

Malaria is a vector-borne infectious disease caused *Plasmodium* parasites, transmitted to humans through the bite of female *Anopheles* mosquitos [110,111]. Five *Plasmodium* species can cause malaria infection in humans: *P. malariae*, *P. falciparum*, *P. vivax*, *P. ovale*, and *P. knowlesi*, with *P. falciparum* and *P. vivax* being the most notorious [15,112]. Malaria is a climate-dependent disease and frequent rainfall provides adequate conditions for mosquito-breeding sites, while temperature affects the development and aggressiveness [111]. The Croatian littoral was once the ideal site for the mosquito reproduction cycle and spreading of malaria due to sufficient rainfall and climate conditions, e.g., the average annual rainfall in the Dalmatia region is between 870 and 980 mm, mostly during winter, enabling the mosquito breeding sites to be active during the whole year [113]. Furthermore, the Slavonia region has also been affected by malaria in the past due to the great number of marshes covering the area [15]. Across endemic regions of Croatia, several members of the *Anopheles* genus are distributed: *An. algeriensis*, *An. claviger*, *An. maculipennis*, and *An. plumbeus* [113,114]. 

The first written records regarding malaria in Croatia were the Statue of the city of Korčula from 1265 [15] and the 16th-century documents from the Istrian peninsula [115,116]. There is a historical reference from 1348 about the sparse population in the city of Nin due to unhealthy air and high mortality, presumably due to malaria. Malaria fever was also described in the city of Novigrad in 1648 and in 1717 in the Istrian town of Dvigrad, which was abandoned due to disease [117]. The first studies on malaria in Croatia were conducted by Giuseppe Antonio Pujati in 1747 [116], and in 1789, the Austrian government was informed about malaria in Istria [15]. There are also records of epidemics in southern Istria and the city of Pula in 1879 [118]. Some authors reported that at the beginning of the 20th century, approximately 180,000 or one-third of the Dalmatia population at that time was affected by malaria [117,119]. The first significant results in the campaign against malaria were achieved during that period after the arrival of Robert Koch to the islands of Brijuni [116]. Koch spent two years (1900–1902) on the islands of Brijuni and successfully eradicated the disease [120]. In 1902, malaria was also eradicated from the town of Nin, and in 1905, it was estimated that around 80,000 individuals were affected by malaria with prevalence varying between 29% in the city of Knin to 63% in the city of Zadar. Malaria was also a notable public health problem on the islands of Krk, Rab, and Pag. From 1922 to 1927, on the island of Krk, gambusia fish were introduced as a successful solution in the anti-malaria campaign [115]. According to a comprehensive review (2019), in the continental region of Slavonia, malaria was discovered in the 18th century by Friedrich Wilhelm von Taube, and the cities of Osijek and Petrovaradin were the most affected. Von Taube stated that Slavonia during that time was covered in unhealthy air and marshes with numerous insects, including mosquitoes. During the same period, the leadership of the Habsburg Monarchy decided to start drying out the marshes and cultivating the fields. In the 19th century, malaria was also present in Lipik, Daruvar, and Virovitica cities [15]. Andrija Štampar and later Branko Richter played a major role in the eradication of malaria during the 20th century, and since 1947, DDT has been used throughout the former Yugoslavia. Since the 1950s, there have been no autochthonous cases of malaria in Croatia, and the disease was officially eradicated in 1964 [15,110,113,121,122].

Since the eradication of the disease, there have been 423 cases of malaria, all imported [15]. Malaria cases in Croatia were retrospectively evaluated from 1987 to 2006 [123]. There were 201 registered cases with a mean of 10.0 ± 2.6 cases annually. Areas of exposure were predominantly Africa (79.6%) and Asia (17.4%) by *P. falciparum* (64.7%) and *P. vivax* (19.9%). Among the infected population, the most numerous were domestic or foreign seafarers. Employed seafarers from the one Croatian sea carrier were specifically investigated in the 4-year period (1990–1993) with the detection of 23 malaria cases, 19 among sailors and 4 among tourists [124]. During 2007, there were 8 identified cases of malaria, all imported from Africa, and 4/8 due to transmission of *P. falciparum* [122]. In a recent study (2021), the authors investigated the incidence of malaria in the period from 2004 to 2014. A total of 102 malaria cases were detected, predominantly in men (89) and skilled workers, university degree workers, and seafarers (30.39, 24.51, and 24.51%, respectively). Patients were mostly infected by *P. falciparum*, and one was deceased [110]. 

## 6. Monitoring of Invasive Mosquito Species in Croatia

Fifty-two mosquito species have been detected in Croatia so far, wherein only a few species have considerable vector potential [125]. The mosquito monitoring system represents an important part of the response to vector-borne diseases. Activities such as human health surveillance, reservoir, and vector control are the most crucial components in risk assessment and management of health threats to both humans and animals. Among different species inhabiting the territory of Croatia, invasive mosquito species have received attention in recent years. In particular, the Asian tiger mosquito, *Aedes* (*Stegomyia*) *albopictus* (Skuse, 1894), which was first discovered in Zagreb in 2004, has received special interest [126]. This species was discovered and afterward established in northwestern and eastern continental regions as well as several locations in coastal Croatia and islands over the next years [125,127,128]. It is a species that shows high vector potential of DENV, CHIKV, and ZIKV [129]; however, it is able to transmit WNV and USUV as well [130]. Following the Croatian Institute of Public Health initiative, national monitoring of invasive mosquito species in Croatia was established in 2016 and continuously performed thereafter. The monitoring has been performed using the ovitraps method at potential entrance points for invasive mosquito species (cemeteries, gas stations, tire storage locations, tire repair shops, truck parking places, border crossings) and house yards. The monitoring revealed another invasive species, *Aedes* (*Finlaya*) *japonicus* (Theobald, 1901), recorded for the first time in September 2013 in Đurmanec and the Macelj border crossing zone (Krapina-Zagorje County) near Slovenia in northwest Croatia. Over the next two years, this species developed in bordering regions in sufficient numbers to allow for its detection as early as early May and as late as October. In addition, in 2015, *Ae. japonicus* was discovered around 100 km east of its initial detection location in 2013. Its expansion was confirmed in 14 Croatian counties [131]. Additionally, in several counties, monitoring of native mosquito species (*Culex*, *Anopheles*, *Ochlerotatus*) has been conducted.

**Figure 9:**
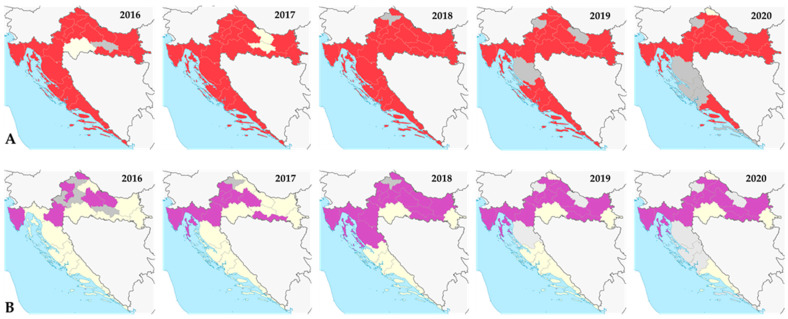
Distribution of *Aedes albopictus* (**A**) and *Aedes japonicus* (**B**) in Croatia, 2016–2020. (**A**): red shadowed areas represent counties with positive records, yellow shadowed areas with negative records, and gray shadowed areas—no data. (**B**): Purple shadowed areas represent counties with positive records, yellow shadowed areas with negative records, and gray shadowed areas—no data.

The presence and spreading of invasive mosquito species (*Ae. albopictus* and *Ae. japonicus*) reported during the national monitoring of invasive mosquito species in the period from 2016 to 2020 are presented in Figure 9. 

*Aedes (Stegomyia) flavopictus* (Yamada, 1921) is an invasive mosquito species closely related to *Ae. albopictus*, which was recently introduced in Europe (the Netherlands, June 2019) [132]. Although it is widely distributed in Japan and South Korea, this species has not been previously recorded outside its native range. In contrast to *Ae. albopictus*, *Ae. flavopictus* is not known as a disease vector in the field; however, this species has shown vector competence for DENV in laboratory conditions. Its discovery highlights the importance of active mosquito surveillance programs in risk areas for timely intervention against exotic mosquito species that could have a negative impact on public health [132,133].

## 7. Conclusions and Future Perspectives

Vector-borne diseases have increased in incidence over the last several decades and represent a significant threat to global health. As a result of changes in modern lifestyle, the number of outbreaks and geographic range of new and re-emerging zoonoses will continue to rise. In addition, climate change has already had an impact on the spread of a variety of vector-borne diseases and will do so going forward since the majority of vector-borne and zoonotic diseases are climate sensitive. According to climate change predictions, there could be a shift in the distribution vector and host populations as a result of increased winter temperatures, longer growth seasons, and earlier summers with elevated temperatures. Ticks are sensitive to environmental factors, particularly temperature and humidity and there has been a documented expansion to higher latitudes and altitudes [134]. Climate change is also believed to have been a factor that influences mosquito dispersal. In France, the Balkans, the eastern coasts of Spain, the Adriatic Sea, and western Germany, the introduction and geographic spread of *Ae. albopictus* has coincided with favorable meteorological conditions [135]. Highly elevated summer temperatures in 2018 have been associated with the largest outbreak of WNV in Europe, including Croatia. In addition to the extent of the outbreak, the early start (end of May) and the extension of transmission season to the end of November were observed that year [136].

Continuous monitoring and evaluation of risk factors associated with vectors, diseases, and human health are required to protect the population from vector-borne diseases. Monitoring weather forecasts can help identify precursors of epidemics and act as an early warning system for risk reduction of vector-borne diseases [134]. Several activities, including vector surveillance, entomological activity, early detection systems, preventive measures, counter-epidemic measures, and viral detection in vectors, reservoirs, or infected animals, must be integrated and considered in order to address these issues, highlighting the importance of multisectoral collaboration between human and veterinary medicine, biology, entomology, forestry, and the meteorology sector (‘One Health’ concept) [137]. 

Since Croatia in the past two decades has faced several (re-)emerging arboviruses (DENV, WNV, USUV), and given that we are surrounded by countries that consistently report the same issues, it is crucial to strengthen mosquito control measures by conducting a nationwide needs assessment. Even though monitoring and control of vectors are regulated in Croatia, there are still shortcomings in practice, as it has been observed that only a few local self-government units are conducting mandatory preventive measures to suppress vectors as required by law and profession [132]. In addition to mosquitoes, it is also important to establish national monitoring of ticks and sandflies due to their vector role for different bacteria, viruses, and parasites. All concerned professionals have a responsibility to ensure successful prevention and public health protection in the modern world by implementing the ‘One Health’ approach into daily practice.

However, structural issues causing collapsing health ecologies have not been addressed by ‘One Health’ research. The ‘Structural One Health’ scope is the interaction between ‘One Health’ science and its political economy, in particular the conceptual and methodological paths via which it fails to incorporate the socioeconomic determinants of epizootic spillover. ‘Structural One Health’ incorporates all sources of cause and effect, including episodic situations, fundamental and historical backgrounds, and the scientific practice itself. Other structural approaches to multispecies health need further investigation [138].

## Figures and Tables

**Table 1:** Sampling sites of *Haemaphysalis punctata* Canestrini and Fanzago, 1878 in Croatia.

Locality	Altitude-Latitude (Degree/min/s)	UTM
1. Bale	N 45°02′22″ E 13°47′09″	VK 08
2. Biokovo Mt.	–	XH 79
3. Brač Island:		XH 49
Blato	N 43°17′02″ E 16°40′41″	
Osridke	N 43°16′55″ E 16°49′24″	
Sveti Toma	N 43°17′34″ E 16°47′00″	
4. Brač Island:		XH 59
Laščatna (North)	N 43°19′01″ E 16°53′25″	
Laščatna (West)	N 43°19′02″ E 16°53′14″	
5. Brač Island (Supetar)	N 43°23′00″ E 16°33′23″	XJ 20
6. Cres Island:		
Belej	N 44°46′41″ E 14°25′46″	VK 56
Hrasta	N 44°48′50″ E 14°25′14″	
7. Cres Island:		VK 57
Cres	N 44°57′38″ E 14°24′33″	
Vrana	N 44°50′40″ E 14°26′32″	
8. Cres Island (Vodice)	N 45°00′31″ E 14°23′58″	VK 58
9. Donji Miholjac	N 45°45′44″ E 18°09′55″	BR 87
10. Dubrovnik	N 42°40′06″ E 18°01′45″	BN 62
11. Goričan	N 46°22′59″ E 16°40′45″	XM 23
12. Hvar Island	–	XH 18
13. Kaštela	N 43°33′09″ E 16°20′36″	XJ 12
14. Koprivnica	N 46°09′50″ E 16°50′00″	XM 41
15. Krk Island (Njivice)	N 45°09′48″ E 14°32′32″	VL 60
16. Lošinj Island:		
Ćunski-Like	N 44°35′21″ E 14°24′35″	VK 63
Veli Lošinj	N 44°31′09″ E 14°30′09″	
17. Metković	N 43°03′12″ E 17°38′57″	YH 06
18. Ponikve (Bakar)	N 45°10′22″ E 14°52′53″	VL 61
19. Požega	N 45°19′53″ E 17°40′28″	YL 12
20. Primišlje	N 45°10′55″ E 15°28′22″	WL 30
21. Pula	N 44°51′59″ E 13°50′58″	VK 16
22. Sinj	N 43°42′10″ E 16°38′15″	XJ 34
23. Gumance (Smrekova Draga)	N 45°30′23″ E 14°25′02″	VL 64
24. Split	N 43°30′29″ E 16°26′25″	XJ 11
25. Sveti Juraj (Senj)	N 44°55′42″ E 14°55′13″	VK 98
26. Kurjak (Udbina)	N 44°29′34″ E 15°39′24″	WK 63
27. Virovitica	N 45°49′54″ E 17°23′08″	XL 87
28. Zadar	N 44°07′11″ E 15°13′59″	WJ 18
29. Zagreb–Karlovac–Sisak (Petrinja)–Bjelovar (Gornja Posavina)	–	WL 75
30. Muć	N 43°41′29″ E 16°32′29″	XJ 13

UTM = Universal Transverse Mercator coordinate system.

**Table 2:** *A. phagocytophilum* seropositivity rates among Croatian dog population.

Location (Year)	N Positive /Tested Dogs	Seropositivity % (95% CI)	Reference
Continental, coastal (2017)	27/435	6.21 (4.13–8.90)	[88]
Continental, coastal (2017)	3/1080	0.28 (0.06–0.81)	[81]
Continental, coastal (2019)	64/1433	4.47 (3.46–5.76)	[76]
Coastal; Istria (2019)	5/134	3.73 (1.22–8.49)	[89]

## Data Availability

Not applicable.

## Supplementary Materials

The following supporting information can be downloaded at: https://www.mdpi.com/article/10.3390/life13091856/s1, Table S1: Sampling sites of *Ixodes ricinus* (Linnaeus, 1758) in Croatia.
