# Supplementary material for: Current Status of Vector-Borne Diseases in Croatia: Challenges and Future Prospects
Source: Life (Basel). 2023 Sep 1;13(9):1856. doi: 10.3390/life13091856 (PMC10532474; doi:10.3390/life13091856)
Supplement: Supplementary file 1 [file life-13-01856-s001.zip › life-2562390-supplementary.pdf]

Table S1. Sampling sites of *Ixodes ricinus* (Linnaeus, 1758) in Croatia.

Locality	Altitude-latitude (degree/min/sec)	UTM
1. Bale	N 45°02'22'' E 13°47'09''	VK 08
2. Baćinska jezera	N 43°04'39'' E 17°24'54''	XH 97
3. Banjol Island	N 45°04'21'' E 13°36'35''	UK 99
4. Batina	N 45°50'29'' E 18°50'44''	CR 38
5. Beli Manastir	N 45°46' 06'' E 18°37' 47''	CR 17
6. Bilje	N 45°36' 16'' E 18°44' 39''	CR 25
7. Bidrovec	N 45°53' 41'' E 16°01' 47''	WL 77
8. Bjelovar	N 45°53'55'' E 16°50'32''	XL 48
9. Biograd-Vrana	N 43°56'09'' E 15°26'33''	WJ 36
10. Biokovo Mt.	-	XH 79
11. Brač Island	N 43°18'17'' E 16°39'09''	XJ 30
12. Branjina	N 45°49'22'' E 18°41'35''	CR 27
13. Brijuni	N 44°56'20'' E 13°40'20''	VK 07
14. Budakovac	N 45°51'03'' E 17°38'16''	XL 97
15. Cres Island:<br>Belej<br>Hrasta	 N 44°46'41'' E 14°25'46''<br>N 44°48'50'' E 14°25'14''	 VK 56
16. Cres Island:<br>Cres<br>Vrana	 N 44°57'38'' E 14°24'33''<br>N 44°50'40'' E 14°26'32''	 VK 57
17. Cres Island:<br>Vodice	 N 45°00'31'' E 14°23'58''	 VK 58
18. Cugovec	N 45°54'28'' E 16°30'56''	XL 18
19. Čakovec	N 46°23'24'' E 16°26'17''	XM 13
20. Čeminac	N 45°41'12'' E18°40'6''	CR 16
21. Dalj Planina	N 45°31'09'' E18°57'54''	CR 00
22. Dekanovec	N 46°26'48'' E 16°34'49''	XM 24
23. Delnice	N 45°23'55'' E 14°48'05''	VL 82
24. Donje Tihovo	N 45°25'59'' E 14°50'49''	VL 82
25. Donji Andrijevci	N 45°11'19'' E 18°17'50''	BR 80
26. Dotrščina	N 45°51'06'' E 16°01'12''	WL 77
27. Draž	N 45°50'30'' E 18°47' 8''	CR 27
28. Duboševica	N 45°53'05'' E 18°41'46''	CR 28
29. Dubrovnik	N 42°39'02'' E 18°05'40''	BN 62
30. Dugo Selo	N 45°48'21'' E 16°14'16''	WL 97
31. Duzluk	N 45°30'55'' E17°51'53''	YL 24
32. Đakovo	N 45°18'25'' E 18°25'01''	BR 92
33. Erdut	N 45°31'29'' E 19°03'36''	CR 44
34. Figarola Island	N 45°05'41'' E 13°37'17''	UK 99
35. Garešnica	N 45°34'27'' E 16°56'26''	XL 54
36. Grabovac	N 45°41'42'' E 18°44'34''	CR 26
37. Grujica Island	N 44°24'37'' E 14°34'09''	VK 62
38. Gumance	N 45°30′23″ E 14°25′02″	VL 64
39. Gustinja Island	N 45°00'59'' E 13°40'53''	UK 99
40. Haljevo (Beli Manastir)	N 45°44'27'' E 18°36'42''	CR 16

41. Haljevo (Darda)	N 45°37'30'' E 18°41'21''	CR 25
42. Ilovik Island	N 44°27'09'' E 14°33'05''	VK 62
43. Ivanovo Selo	N 45°40'25'' E 17°15'02''	XL 76
44. Kalnik	N 46°07'50'' E 16°27'16''	XM 21
45. Kaštela	N 43°33'51'' E 16°21'29''	XJ 02
46. Koprivnica	N 46°09'50'' E 16°50'00''	XM 41
47. Koška	N 45°32'44'' E 18°16'59''	BR 84
48. Koversada	N 45°08'03'' E 13°35'51''	UL 90
49. Kozjak mountain: Malačka	N 43°34'44'' E 16°21'04''	XJ 02
50. Krk Island	N 45°00'38'' E 14°45'24''	VK 79
51. Krndija (ustava)	N 45°27'48'' E 18°23'09''	BR 93
52. Kunjevci	N 45°13'53'' E 18°48'04''	CR 21
53. Kurjak reef	N 44°24'17'' E 14°45'06''	VK 71
54. Lakal Island	N 45°09'41'' E 13°35'33''	UL 90
55. Lika: Plitvice-Crna rijeka	N 44°50'41'' E 15°37'45''	WK 46
56. Lipik	N 45°24'51'' E 17°09'40''	XL 63
57. Lipovljani	N 45°23'53'' E 16°53'40''	XL 42
58. Lošinj Island: Veli Lošinj	N 44°31'09'' E 14°30'09''	VK 63
59. Lovas	N 45°13'34' E 19°10'07''	CR 50
60. Lučice, Delnice	N 45°22' 26'' E 14°48' 02''	VL 82
61. Lug	N 45°39'48'' E 18°46'26''	CR 25
62. Lukovdol- Lipov vrh	N 45°25'33'' E 15°07'37''	VL 03
63. Lunga Island	N 45°08'32'' E 13°34'58''	UL 90
64. Male Orjule Island	N 44°29'20'' E 14°33'53''	VK 62
65. Marija Trošt	N 45°25'12'' E 14°49'27''	VL 82
66. Markovac	N 45°25'58'' E 17°32'22''	YL 23
67. Medvednica	N 45°53'20'' E15°58'33''	WL 78
68. Milivojevci, Požega	N 45°25' 40'' E 17°31' 14''	YL 12
69. Metković-Gabela	N 43°04'01'' E 17°39'08''	YH 16
70. Mikleuš	N 45°36'46'' E 17°48'33''	YL 15
71. Nard	N 45°39'51'' E 18°28'49''	CR 06
72. Nedelišće	N 46°22'34'' E 16°23'08''	XM 12
73. Nemetin	N 45°32'23'' E 18°46'15''	CR 24
74. Normanci	N 45°33'21'' E 18°21'44''	BR 94
75. Nova Gradiška	N 45°15'29'' E 17°23'02''	XL 81
76. Novigrad na Dobri	N 45°28'19'' E 15°26'31''	WL 33
77. Ogulin	N 45°15'57'' E 15°13'25''	WL 11
78. Orahovica	N 45°31'44'' E 17°52'49''	YL 24
79. Oruda Island	N 44°33'07'' E 14°34'57''	VK 63
80. Osijek	N 45°33' 17'' E 18°41' 43''	CR 24
81. Otok	N 45°08'47'' E 18°53'02''	CR 30
82. Palacol Island	N 44°32'40'' E 14°35'39''	VK 63
83. Peščenica-Vratovo	N 45°36'08'' E 16°10'05''	WL 95
84. Petrijevci	N 45°36'51'' E 18°32'12''	CR 05
85. Petrinja	N 45°25'31'' E 16°14'48''	WL 93
86. Pisulj Island	N 45°01'12'' E 13°41'51''	UK 99
87. Piškorevci	N 45°15'16'' E 18°23'57''	BR 91
88. Podolje	N 45°48'58'' E 18°43'43''	CR 27

89. Podravlje (Osijek)	N 45°33'53'' E 18°42'30''	CR 24
90. Podravska Moslavina	N 45°47'07'' E 17°58'55''	YL 37
91. Poganovci	N 45°29'22'' E 18°23'59''	BR 94
92. Polonje, Sveti Ivan Zelina	N 45°57'49'' E 16°16'46''	WL 99
93. Ponikve (Bakar)	N 45°10'22'' E 14°52'53''	VL 61
94. Popovac	N 45°48'22'' E 18°39'34''	CR 17
95. Popovača	N 45°34'16'' E 16°37'38''	XL 24
96. Poreč	N 45°13'37'' E 13°35'41''	UL 90
97. Primišlje	N 45°10'55'' E 15°28'22''	WL 30
98. Pula	N 44°51'59'' E 13°50'58''	VK 16
99. Pulari Island	N 45°03'25'' E 13°39'51''	UK 99
100. Punta Križa	N 44°38'15'' E 14°29'39''	VK 54
101. Pušća	N 45°55'01'' E 15°46'10''	WL 68
102. Puščine	N 46°21' 07'' E 16°22'10''	XM 03
103. Radovanci	N 45°30'67'' E 17°39'43''	YL 03
104. Rečica	N 45°30'27'' E 15°39'46''	WL 53
105. Revera Island	N 45°03'02'' E 13°39'34''	UK 99
106. Rovinj	N 45°04'52'' E 13°38'19''	UK 99
107. Saborsko	N 45°02'29'' E 15°19'16''	WK 38
108. Senj	N 44°59'21'' E 14°54'12''	VK 98
109. Seona	N 45°28'24'' E 18°01'28''	BR 74
110. Sisak	N 45°29'06'' E 16°22'23''	XL 03
111. Skrad	N 45°25'45'' E 14°54'54''	VL 93
112. Slatinski Drenovac	N 45°31'55'' E 17°41'30''	YL 14
113. Slunj	N 45°06'53'' E 15°35'16''	WK 49
114. Sljeme	N 45°53'57'' E 15°56'51''	WL 78
115. Sotin	N 45°17'46'' E 19°05'48''	CR 51
116. Spačva	N 45°03'15'' E 19°01'48''	CQ 38
117. Split	N 43°30'29'' E 16°26'25''	XJ 11
118. Srednja Posavina: Stružec	N 45°31'37'' E 16°32'59''	XL 24
119. Stara Ves	N 45°58'35'' E 16°29'56''	XL 39
120. Stari Laz	N 45°21' 40'' E 14°52' 39''	VL 92
121. Sturag Island	N 45°03'05'' E 13°37'34''	UK 99
122. Sunger	N 45°19'21'' E 14°49'11''	VL 81
123. Sungerski Lug	N 45°19'22'' E 14°49'12''	VL 81
124. Susak Island	N 44°30'28'' E 14°18'14''	VK 42
125. Sveti Đurađ	N 45°44'24'' E 18°14'33''	BR 86
126. Sveti Ivan Island	N 45°02'49'' E 13°37'17''	UK 99
127. Sveti Ivan np reef	N 45°02'33'' E 13°36'50''	UK99
128. Sveti Ivan Žabno	N 45°56'43'' E 16°36'20''	XL 29
129. Sveti Petar Island	N 44°27'43'' E 14°33'29''	VK 62
130. Široko Polje	N 45°24'14'' E 18°28'20''	CR 03
131. Školjić reef	N 44°38'09'' E 14°13'54''	VK 54
132. Tikveš	N 45°40'20'' E 18°50'37''	CR 36
133. Tompojevci	N 45°13'57'' E 19°05'32''	CR 41
134. Topoline	N 45°33'41'' E 18°22'33''	BR 84
135. Tovarjež Island	N 45°10'07'' E 13°34'55''	UL 90
136. Trakoščan	N 46°15'32'' E 15°56'48''	WM 72
137. Unije Island	N 44°37'29'' E 14°15'34''	VK 34
138. Vele Srakane Island	N 44°34'51'' E 14°18'41''	VK 42
139. Velika	N 45°27'20'' E 17°39'45''	YL 03
140. Velika Gorica	N 45°42'51'' E 16°04'30''	WL 76

141. Velika Kapela mountain: Razvala	N 45°04'35'' E 15°12'41''	WK 29
142. Velika Kopanica	N 45°09'21'' E 18°23'40''	BR 90
143. Veliki Piruzi reef	N 45°03'32'' E 13°38'22''	UK 99
144. Velika Sestrica Island	N 45°02'00'' E 13°40'28''	UK 99
145. Veštar Island	N 45°02'51'' E 13°40'42''	UK 99
146. Viljevo	N 45°45'05'' E 18°03'48''	BR 77
147. Vinkovci	N 45°17'16'' E 18°48'20''	CR 21
148. Visuć (Udbina)	N 44°31' 35'' E 15°50' 07''	WK 63
149. Voćin	N 45°37' 11'' E 17°32' 40''	XL 95
150. Vrbanja	N 45°00' 1'' E 18°51'09''	CQ 38
151. Vrpolje	N 45°12'37'' E 18°24'19''	BR 90
152. Vukomeričke Gorice: Prkovec	N 45°37'00'' E 15°56'59''	WL 84
153. Vukovar	N 45°20'43'' E 19°00'04''	CR 42
154. Zadar	N 44°07'11'' E 15°13'59''	WJ 18
155. Zagreb	N 45°48'47'' E 15°58'40''	WL 77
156. Zagreb–Karlovac –Sisak (Petrinja) –Bjelovar (Gornja Posavina)	-	WL 75
157. Zeča Island	N 44°46'20'' E 14°18'41''	VK 56
158. Zlatna Greda (Kopački rit NP)	N 45°43'16'' E 18°51'49''	CR 36
159. Zmajevac	N 45°47'59'' E 18°48'23''	CR 37
